# Work Ability, Burnout Complaints, and Work Engagement Among Employees With Chronic Diseases: Job Resources as Targets for Intervention?

**DOI:** 10.3389/fpsyg.2020.01805

**Published:** 2020-08-06

**Authors:** Ingrid G. Boelhouwer, Willemijn Vermeer, Tinka van Vuuren

**Affiliations:** ^1^Department of Applied Psychology, Amsterdam University of Applied Sciences, Amsterdam, Netherlands; ^2^Faculty of Management, Open University of The Netherlands, Heerlen, Netherlands; ^3^Loyalis Knowledge & Consult, Heerlen, Netherlands

**Keywords:** work ability, burnout complaints, work engagement, chronic diseases, multimorbidity, occupational well-being, job resources

## Abstract

**Purpose:**

The aim of this study was to investigate the occupational well-being among employees with chronic diseases, and the buffering effect of four job resources, possibly offering targets to enhance occupational well-being.

**Method:**

This cross-sectional study (*N* = 1951) was carried out among employees in educational and (semi-)governmental organizations in the Netherlands. The dimensions of the survey were chronic diseases (i.e., physical, mental, or both physical and mental), occupational well-being (i.e., work ability, burnout complaints, and work engagement), and job resources (i.e., autonomy, social support by colleagues, supportive leadership style, and open and communicative culture). First, it was analyzed if chronic diseases were associated with occupational well-being. Second, it was analyzed if each of the four job resources would predict better occupational well-being. Third, possible moderation effects between the chronic disease groups and each job resource on occupational well-being were examined. Regression analyses were used, controlling for age.

**Results:**

Each chronic disease group was associated with a lower work ability. However, higher burnout complaints and a lower work engagement were only predicted by the group with mental chronic diseases and by the group with both physical and mental chronic disease(s). Furthermore, all four job resources predicted lower burnout complaints and higher work engagement, while higher work ability was only predicted by autonomy and a supportive leadership style. Some moderation effects were observed. Autonomy buffered the negative relationship between the chronic disease groups with mental conditions (with or without physical conditions) and work ability, and the positive relationship between the group with both physical and mental chronic disease(s) and burnout complaints. Furthermore, a supportive leadership style is of less benefit for occupational well-being among the employees with mental chronic diseases (with or without physical chronic diseases) compared to the group employees without chronic diseases. No buffering was demonstrated for social support of colleagues and an open and communicative organizational culture.

**Conclusion:**

Autonomy offers opportunities to reinforce occupational well-being among employees with mental chronic diseases. A supportive leadership style needs more investigation to clarify why this job resource is less beneficial for employees with mental chronic diseases than for the employees without chronic diseases.

## Introduction

A chronic disease is regarded as a disease with an episode of treatment that extends over a long period, although the condition or stage of the disease does not have to be serious (De Lepeleire and Heyrman, 2003). Examples of chronic diseases are musculoskeletal diseases, cardiovascular diseases, or depression. The labor market participation of the population with chronic diseases is lower than that of the population without chronic diseases. For the general population at working age with chronic diseases in the Netherlands, the labor market participation (for at least 12 h a week) is lower than that of the population without one or more chronic diseases, namely, 25% and 67%, respectively, in 2010 (Maurits et al., 2013). Furthermore, the labor market participation of the group with chronic diseases in the Netherlands is comparable to the mean figure of the other member countries of the Organization for Economic Cooperation and Development (SER, 2016).

The need to find strategies and solutions for enhancing the employment of people with chronic diseases is widely acknowledged (Nazarov et al., 2019). However, a factor in the prevalence of chronic diseases among workers is age. The last decades, the expansion of the aging workforce is an important factor among others with regard to the preservation of productivity (Aiyar and Ebeke, 2016), job opportunities, careers, and social inclusion (Silvaggi et al., 2020). The prevalence of one or more chronic diseases in the Netherlands in the year 2018 was 20.7% among the group in the age category from 20 to 30 years old, up to 46.4% among the group in the age category from 55 to 65 years old (CBS, 2019). Furthermore, comorbidity and multimorbidity of various chronic diseases is expected to continue to increase (Uijen and van de Lisdonk, 2008; Boyd and Fortin, 2010). As a consequence, the number of people at risk of experiencing difficulties with particular work activities or demands as a result of one or more chronic diseases will be even more substantial in the future. Therefore, it is increasingly important to focus on working people with chronic diseases and indicate possible targets to enhance their occupational well-being.

Occupational well-being can be regarded as a broad concept, including work ability, burnout complaints, and work engagement, which are the indicators in the present study. Work ability, the first indicator, refers to one’s ability to function well at work or to be able to achieve expected work goals (Ilmarinen et al., 2005; Ilmarinen, 2007). Work ability is mostly measured by one or more items of the Work Ability Index (WAI) questionnaire (Ilmarinen, 2007). The level of work ability is regarded as a valid indicator for other work outcome measures. For instance, a moderate or poor work ability is found to be highly predictive for receiving a disability pension (Alavinia et al., 2009). Although higher age is associated with more chronic diseases, studies do report mixed results with regard to the association between age and work ability. Some studies report a decreased work ability with older age (van den Berg et al., 2008; Vangelova et al., 2018); however, also high work ability among older workers is reported, for instance, in an Australian study among mature age working women (Austen et al., 2016). Burnout complaints, the second indicator, are regarded as a prolonged stress response to chronic stressors at work, which might be related to the onset of cognitive decline in elderly workers (Giorgi et al., 2020). Burnout can be defined by three dimensions; exhaustion, cynicism, and inefficacy (Maslach et al., 2001), which distinction is in line with the subscales of the Utrecht Burnout Scale (UBOS) (Schaufeli and van Dierendonck, 2000). Work engagement, the third indicator, is described as a positive, fulfilling, affective-motivational state of work-related well-being that is characterized by vigor, dedication, and absorption, in line with the subscales of the Utrecht Work Engagement Scale (UWES) (Bakker et al., 2008).

Furthermore, supporting factors in achieving work goals, so-called job resources within the Job Demands-Resources (JD-R) model (Demerouti et al., 2001), might play an important role. Job resources refer to aspects of the job that are functional in achieving work goals, or stimulate personal growth, learning and development or reduce job demands (Schaufeli and Bakker, 2004). In the JD-R model, job demands are regarded as the aspects of the job that require effort and it is possible that the effects of a chronic disease result in work demands being experienced as heavier. In general, the JD-R model and therefore the beneficial influence of job resources is well established in several work contexts. In addition, in some studies, job resources were reported to buffer the impact of job demands on burnout (Bakker et al., 2005; Xanthopoulou et al., 2007b). So, job resources can show positive associations with higher occupational well-being, or job resources might even buffer a possible association between chronic diseases and occupational well-being. For that reason, job resources might be of importance for work functioning among workers with chronic diseases.

Research on associations among the three indicators of occupational well-being as used in this study is merely focused on associations between burnout complaints and work engagement. Studies indicate that these two indicators of occupational well-being cannot simply be regarded as opposite concepts. Burnout is mainly predicted by job demands and a lack of job resources. However, work engagement is specifically predicted by available job resources (Schaufeli and Bakker, 2004). Studies on relations between burnout complaints or work engagement on the one hand and work ability on the other hand are scarce. Nevertheless, there are some results. For instance, in a 6-month longitudinal study among employees of two manufacturers Rongen et al. (2014) demonstrated that low work engagement was related with low work ability beyond known health behaviors and work-related characteristics. These findings indicate that it is important to examine all three abovementioned indicators of occupational well-being within one study.

## Hypotheses

The aim of the present study is to investigate (1) if chronic diseases are associated with lower occupational well-being (that is, lower work ability, higher burnout complaints, or lower work engagement), (2) the direct effect of four job resources on occupational well-being, and, most importantly, (3) the possible moderation of the presumed relationship between chronic diseases and occupational well-being by job resources. The approach to combine these concepts in one study is quite unique and of great relevance in finding possible targets to enhance occupational well-being among employees with chronic diseases.

### Chronic Diseases and Occupational Well-Being

Chronic diseases are normally categorized as mental or physical. Different chronic diseases can demonstrate various profiles with regard to the mean age at which the condition occurs in the population and the prevalence of the condition between age groups. Furthermore, a higher prevalence of multimorbidity is observed with higher age (Banerjee, 2015; Xu et al., 2017). Therefore, the present study will differentiate between physical chronic diseases, mental chronic diseases, and comorbidities of mental and physical chronic diseases, as these groups might differ with regard to the stage in the course of life and career in which the members of the group find themselves.

Regarding work ability, several studies have already investigated the association of chronic diseases with this indicator of occupational well-being. Workers with different chronic diseases are reported to be at a higher risk of a lower level of work ability than workers without these conditions (Koolhaas et al., 2013; Leijten et al., 2014; van den Berg et al., 2017).

Furthermore, burnout complaints are found to occur more frequently in certain populations with specific chronic diseases, such as women with musculoskeletal diseases or men with cardiovascular diseases (Honkonen et al., 2006), women with coronary heart disease (Hallman et al., 2003) or women with depression (Soares et al., 2007). On the other hand, burnout is also reported to probably influence the development and course of certain disease processes by several biobehavioral pathways (Shirom et al., 2006), but this is only studied for some chronic diseases.

The available studies that assess the association between chronic diseases and work engagement do not directly present an unambiguous overview. Schaufeli et al. (2008) reported significant negative associations between perceived health and dimensions of work engagement. Furthermore, distress and depression were both negatively associated with vigor, and distress was negatively associated with dedication as well. However, in another study, high work engagement levels were observed among workers with musculoskeletal symptoms, but the role of the biomechanical demands of the work tasks of these workers needed further investigation (Nogueira et al., 2012). Furthermore, another study among cancer survivors (after they had returned to work) and their non-cancer referents (with or without other chronic diseases) demonstrated that the level of work engagement was high in both study groups, and only slightly higher among the referents than among the cancer survivors (Hakanen and Lindbohm, 2008). In general, vigor is positively related to mental and physical health (Bakker and Leiter, 2010). Furthermore, vigor is regarded as a physical indicator of vitality (Ryan and Frederick, 1997), and vitality is regarded to be related to the absence of chronic diseases (Strijk et al., 2009).

To summarize, employees with chronic diseases are expected to have lower work ability, higher burnout complaints, and lower work engagement than employees without chronic diseases. Hence, our first set of hypotheses is:

H1a. Workers with chronic diseases (mental and/or physical chronic diseases) have a lower work ability than workers without chronic diseases.
H1b. Workers with chronic diseases (mental and/or physical chronic diseases) have higher burnout complaints than workers without chronic diseases.
H1c. Workers with chronic diseases (mental and/or physical chronic diseases) have lower work engagement than workers without chronic diseases.

### Job Resources and Occupational Well-Being

Assuming that occupational well-being is less favorable in the case of chronic diseases as we expected, it is important to have more insight in possible specific ways to improve occupational well-being by promoting job resources for this population of workers. In the present study, four job resources are taken into account: autonomy, social support by colleagues, a supportive leadership style, and an open and communicative culture. Firstly, in this section, we will formulate our expectations with regard to the association of each of the four job resources with occupational well-being, and then in Section “Moderation by Job Resources,” we will present our expectations on the moderation by the four job resources of the presumed relationship between the chronic disease groups and occupational well-being.

The first job resource, autonomy, refers to the influence on one’s own work, for instance by autonomous decisions. Several studies have demonstrated that a lack of autonomy is associated with poor work ability, as defined by the WAI (van den Berg et al., 2008). Associations of autonomy and burnout complaints were found as well, and a lack of autonomy is correlated with burnout risk (Maslach et al., 2001; Kim et al., 2018). Furthermore, a meta-analysis by Alarcon (2011) demonstrated that autonomy is negatively associated with all three burnout subscales. With regard to work engagement, a cross-national study in different work contexts within eight European countries by Taipale et al. (2011), demonstrated that autonomy was a strong predictor of the level of work engagement. Furthermore, job control is associated with work engagement among Finnish health care personnel in a longitudinal study by Mauno et al. (2007). Schaufeli et al. (2009) reported changes in autonomy to be predictive of changes in work engagement among telecom managers.

With regard to the second job resource, social support by colleagues, a positive association with work ability is demonstrated for instance among hospital nurses (Olsen et al., 2017). Furthermore, among female cancer survivors, co-workers’ support is related to a reduced risk of impaired work ability (Taskila et al., 2007). Concerning burnout complaints, there is a consistent and strong body of evidence that a lack of social support is linked to burnout (Maslach et al., 2001). Also, with regard to work engagement, a positive association of social support by work mates with work engagement is for instance reported in the study in eight European countries in different work contexts (Taipale et al., 2011).

The third job resource, a supportive leadership style, is studied in relation to work ability in previous studies, but results vary. Among IT workers, supervisor support was demonstrated to predict work ability 1 year later (Sugimura and Thériault, 2010). However, in a study by Tuomi et al. (2004), an improvement of supervisory support did not predict an improvement of work ability, although improvement of supervisory support and improvement of work ability were significantly associated. In another study, conducted in several parts of the industrialized world by McGonagle et al. (2014), supervisor support was positively related to work ability in the Australian sample only, and not associated with work ability in the other samples (i.e., United States, United Kingdom, Brazil, Poland, and Croatia). The relation of a supportive leadership style with burnout is also far from straightforward. Kanste et al. (2007) indicate that this relationship is complex, as leadership style tends to be affected by situational factors. However, Maslach et al. (2001) concluded that a lack of support from supervisors is especially detrimental in relation to burnout complaints, even more so than a lack of support from co-workers. With respect to work engagement, a study demonstrated a higher contribution of transformational leadership to work engagement than transactional leadership (Li et al., 2018). As the latter style focuses on performance within existing boundaries, the transformational leadership is more change-oriented and might allow more use of job resources.

With regard to the fourth job resource, an open and communicative organizational culture, there are some studies that focus on concepts linked to organizational culture. For instance, associations with higher work ability were found for good organizational relationships among personnel of nursing homes (Kiss et al., 2014) and for a supportive organizational climate among managers (Feldt et al., 2009). Higher perceptions of ethical culture demonstrated to be associated with lower burnout and higher work engagement (Huhtala et al., 2015). Furthermore, in a review by Wollard and Shuck (2011) concerning the antecedents of work engagement, not only results with regard to local microcultures and management, like psychological climate (Shuck et al., 2011), were reported, but also antecedents at the organizational level, like corporate social responsibility (Davies and Crane, 2010). Furthermore, van Dam et al. (2017) demonstrated that an age-supportive climate is especially important for older employees’ work engagement and affective commitment.

To summarize, autonomy, social support by colleagues, a supportive leadership style, and an open and communicative culture are expected to be associated with a higher work ability, lower burnout complaints, and higher work engagement. Therefore, our second set of hypotheses is:

H2a. Autonomy is associated with higher work ability, lower burnout complaints, and higher work engagement.
H2b. Social support by colleagues is associated with higher work ability, lower burnout complaints, and higher work engagement.
H2c. A supportive leadership style is associated with higher work ability, lower burnout complaints, and higher work engagement.
H2d. An open and communicative organizational culture is associated with higher work ability, lower burnout complaints, and higher work engagement.

### Moderation by Job Resources

The four job resources can be of importance for employees in the general population, and the focus in this study is on the possible interaction of the presumed relationship between the chronic disease groups and occupational well-being. The JD-R model distinguishes a strain process, related to the level of the job demands, and a motivational process, influenced by job resources. Job resources can buffer for demanding work conditions (Bakker and Demerouti, 2007). As workers with chronic diseases might experience their work as more demanding because of these chronic diseases, several job resources might also buffer the association between the chronic diseases and less favorable work ability, burnout complaints, or work engagement. However, to our knowledge, no study has been done to investigate this among employees with chronic diseases. Among the general population, studies with a focus on buffering effects of job resources do not concern work ability, but several studies concern burnout complaints or work engagement. These studies demonstrated the importance of job resources interacting with job demands predicting lower symptoms of burnout (Bakker et al., 2005; Xanthopoulou et al., 2007a) or higher work engagement (Bakker et al., 2007). Because of the rationale of the JD-R model and the above presented results, we expect a moderating effect for the job resources, and our third set of hypotheses is:

H3a. Autonomy buffers the presumed relationship of chronic diseases with lower work ability, higher burnout complaints, or lower work engagement.
H3b. Social support by colleagues buffers the presumed relationship of chronic diseases with lower work ability, higher burnout complaints, or lower work engagement.
H3c. A supportive leadership style buffers the presumed relationship of chronic diseases with lower work ability, higher burnout complaints, or lower work engagement.
H3d. An open and communicative organizational culture buffers the presumed relationship of chronic diseases with lower work ability, higher burnout complaints, or lower work engagement.

## Materials and Methods

### Participants and Procedure

A cross-sectional employee survey was carried out between 2013 and 2017 in The Netherlands by Loyalis Knowledge & Consult among employees working in different primary schools and (semi) governmental organizations (i.e., municipality and regional water authorities) in accordance with relevant institutional and national guidelines. The aim was to offer the employees in the participating organizations information for improving their sustainable employability. The questionnaires of the present study included scales for four job resources (autonomy, social support by colleagues, supportive leadership style, and open and communicative culture). The questionnaires were distributed online, accompanied by an e-mail on behalf of the researchers, stating the relevance and purpose of the study. The respondents were informed that all data would be treated confidentially and that the participation was voluntary. All subjects gave written informed consent in accordance with the Declaration of Helsinki. The participants were predominantly female (61.6%) and with a high educational level (73.1%). The mean age was 46.4 years (*SD* 11.12).

### Measures

Information on chronic diseases was obtained based on the third question of the WAI questionnaire (Ilmarinen, 2007). This WAI question consists of a list of actual physical or mental conditions, for which the respondent can indicate if this is an actual health condition diagnosed by a physician. The possible physical conditions may be an injury caused by an accident, a condition of the musculoskeletal system, cardiovascular disease, respiratory disease, neurological and sensory disease, digestive disease, genitourinary disease, skin disease, metabolic disease, blood diseases, birth defects, or tumors. The possible mental conditions may be depressive complaints or a depressive disorder, tension, anxiety, and insomnia or other mental disorders. For the present study, the condition(s) are classified in three groups and indicated as chronic diseases. Chronic disease group 1 includes participants with one or more physical condition(s) (*N* = 640) which represents 32.8% of the sample. Among these participants, the most frequently reported chronic diseases are “medical condition of the musculoskeletal system” with 25.3% and “cardiovascular disease” with 11.9%. A number of two physical chronic diseases is reported by 9.8% of the respondents and 5.3% reported three or more physical chronic diseases. Chronic disease group 2 includes participants with one or more mental condition(s) (*N* = 36), which represents 1.8% of the sample. Chronic disease group 3 includes participants with one or more physical conditions and one or more mental conditions (*N* = 120). Chronic disease group 3 represents 6.2% of the sample. The remaining participants in the sample have no physical chronic disease, or a mental chronic disease (*N* = 1155, which represents 59.2% of the sample) (see Tables 1, 2).

**TABLE 1:** Groups with chronic diseases and numbers of chronic diseases (*N* = 1951).

**Chronic diseases**	**Number reported**	**%**
**Groups**		
Group 1—physical chronic disease(s)	640	32.8
Group 2—mental chronic disease(s)	36	1.8
Group 3—physical chronic disease(s) and mental chronic disease(s)	120	6.2

**Chronic diseases**		
Medical condition of the musculoskeletal system	493	25.3
Cardiovascular disease	232	11.9
Skin disease	189	9.7
Respiratory disease	183	9.4
Neurological and sensory disease	173	8.9
Metabolic disease	118	6.0
Digestive disease	114	5.8
Injury caused by an accident	110	5.6
Genitourinary disease	64	3.3
Tumors	43	2.2
Birth defects	31	1.6
Blood diseases	26	1.3
Other	70	3.6

Mental chronic diseases	156	8.0
		

**TABLE 2:** Numbers of physical chronic diseases.

**Number of physical chronic diseases**	**Number of participants**	**%**
1	364	18.7
2	192	9.8
3	81	4.2
4	20	1.0
5 or more	18	0.1

Work ability was measured by a combination score of the first two questions from the WAI. The first question of the WAI indicates the current work ability compared with a person’s lifetime best on a scale from 0 (completely unable to work) to 10 (work ability at its best). This item is reported to have a very strong association with the complete WAI (Ahlstrom et al., 2010). The second question consists of two items: current physical work ability and current mental work ability in relation to physical and mental job demands on a scale from 0 (very low) to 5 (very high). For the present study, the three items were merged into one work ability scale from 0 (very low) to 5 (very high), whereby the scale of the first item was adjusted from 0 (completely unable to work) to 10 (work ability at its best) into a scale from 0 (completely unable to work) to 5 (work ability at its best) before merging. The Cronbach’s α of the final work ability scale with three items is 0.74.

Burnout was measured using the UBOS (Schaufeli and van Dierendonck, 2000), consisting of 15 items on a seven-point Likert scale from 1 (never) to 7 (always), covering three subscales, namely, exhaustion, cynicism, and professional inefficacy. In the present study, the total score of the three subscales was used. The Cronbach’s α’s for the three subscales were respectively 0.89, 0.82, and 0.82 and the reliability of the total UBOS scale is 0.89.

Work engagement was measured using the UWES (Bakker et al., 2008) consisting of nine items on a seven-point Likert scale from 1 (never) to 7 (always), covering three subscales, namely, vigor, dedication, and absorption. In the present study, the total score of the three subscales was used. The Cronbach’s α’s for these three subscales were respectively 0.90, 0.93, and 0.84 and the reliability of the total UWES scale is 0.95.

The four job resources (autonomy, social support by colleagues, a supportive leadership style, and an open and communicative organizational culture) were measured using one of the scales by Van Poppel and Kamphuis (2004), which were developed for the context of primary schools. All four job resources made use of a five-point scale of 1 (strongly disagree), 2 (disagree), 3 (not agree, nor disagree), 4 (agree), and 5 (strongly agree). The Cronbach’s α’s were 0.80 for autonomy (four items), 0.74 for social support by colleagues (five items), 0.89 for supportive leadership style (four items), and 0.90 for open and communicative culture (eight items).

Control variable is calendar age, as reported by the respondents. Age is reported to show different relationships with various chronic diseases and also to be negatively associated with work ability in other studies (van den Berg et al., 2008).

### Analysis

The data were analyzed using SPSS software, version 25 (IBM Corporation, Armonk, NY, United States) for Windows^®^/Apple Mac^®^. Descriptives are reported for age, work ability, burnout complaints, work engagement, and the four job resources (autonomy, social support by colleagues, a supportive leadership style, and an open and communicative organizational culture). The scores on job resources were standardized by *z* scores. For the regression analyses, we used dummies for three categories of employees with chronic diseases (groups 1, 2, or 3), in order to establish the relationship with the type of chronic disease. In doing so, we used employees without chronic diseases (group 4) as the reference category. Hardy (1993) recommends to use a reference category that serves as a useful comparison to the other categories, and to use a large group as the reference category. The reference category is omitted from the regression analyses; the standardized coefficient (β) shows the extent to which the other group deviates from the reference group and is regarded as the indicator of the effect size (Ferguson, 2009). Standardized coefficients are more easily comparable, because the variables are standardized to have a mean of 0 and standard deviation of 1. In line with other common effect indices, a β coefficient of 0.2 is regarded as a small effect, a β coefficient of 0.5 is regarded as a medium effect, and a β coefficient of 0.8 is regarded as a large effect (Sullivan and Feinn, 2012).

Three separate multiple regression analyses were used to investigate the associations between the dummies and each of the job resources with respectively work ability, burnout complaints, and work engagement, also including age in each analysis. Furthermore, possible moderation by autonomy, social support by colleagues, a supportive leadership style, and an open and communicative culture were analyzed by interaction terms of each of the chronic disease groups and each of the four job resources. The dummies for the interaction terms of employees without chronic diseases and each of the four job resources are also omitted.

## Results

### Descriptives

The mean age of the group with physical chronic disease(s) was 47.9 years (*SD* 10.81), and significantly higher (*p* < 0.05) than the mean age of the group with mental chronic disease(s) with 42.1 years (*SD* 11.35) and also than the group without chronic diseases with 45.5 years (*SD* 11.25). The mean age of the group with physical and mental chronic disease(s) [with both physical and mental condition(s)] was 47.5 years (*SD* 10.37) (see Table 3).

**TABLE 3:** Health condition groups: age, work ability, burnout complaints, work engagement, and job resources (autonomy, social support by colleagues, a supportive leadership style, or an open and communicative culture).

**Variable M (SD)**	**Missing**	**Group 1—physical chronic disease(s)**	**Group 2—mental chronic disease(s)**	**Group 3—physical chronic disease(s) and mental chronic disease(s)**	**Group 4—no chronic disease(s)**	**Complete study sample**
*N*		640	36	120	1155	1951
Age in years^μ^	34	47.9 (10.81)^#^	42.1 (11.35)	47.5 (10.37)	45.5 (11.25)	46.4 (11.12)
Work ability^μλ^ Five-point scale	–	3.9 (0.51) ^#^	3.6 (0.67) ^#^	3.4 (0.71) ^#^	4.0 (0.50)	3.9 (0.55)
Burnout complaints^μλ^ Seven-point scale	–	2.4 (0.72) ^#^	3.0 (0.92) ^#^	3.2 (0.94) ^#^	2.3 (0.69)	2.4 (0.76)
Work engagement^μλ^ Seven-point scale	–	5.1 (1.05)	4.2 (1.16) ^#^	4.3 (1.11) ^#^	5.1 (1.11)	5.0 (1.11)
Autonomy Five-point scale	6	3.6 (0.65)	3.5 (0.70)	3.5 (0.70) ^#^	3.7 (0.65)	3.7 (0.65)
Social support by colleagues^λ^ Five-point scale	6	4.0 (0.50)	3.9 (0.50)	3.9 (0.61) ^#^	4.0 (0.52)	4.0 (0.52)
Supportive leadership style^λψ^ Five-point scale	11	3.7 (0.76)	3.8 (0.71)	3.3 (0.93) ^#^	3.7 (0.80)	3.7 (0.80)
Open and communicative culture^λψ^ Five-point scale	8	3.4 (0.67)	3.4 (0.58)	3.0 (0.72) ^#^	3.4 (0.67)	3.4 (0.68)

*M, mean; SD, standard deviation; *N*, number of participants. ^μ^Significant difference between group 1 and group 2 at the 0.05 level. ^λ^Significant difference between group 1 and group 3 at the 0.05 level. ^ψ^Significant difference between group 2 and group 3 at the 0.05 level. ^#^Significant difference from group 4 at the 0.05 level.*

Table 3 also shows that the mean level of work ability was significantly higher in the group without chronic diseases (4.0), than in the group with physical chronic disease(s) (3.9) (*p* < 0.05), the group with mental chronic disease(s) (3.6) (*p* < 0.05), and the group with both physical and mental condition(s) (3.4) (*p* < 0.05). Furthermore, the level of work ability in the group with physical chronic disease(s) was significantly higher than in the group with mental chronic disease(s) (*p* < *0.05*) and the group with both physical and mental condition(s) (*p* < *0.05*). The mean level of burnout complaints was significantly lower in the group without chronic diseases (2.3) than in the group with physical chronic disease(s) (2.4) (*p* < *0.05*), the group with mental chronic disease(s) (3.0) (*p* < *0.05*), and the group with both physical and mental condition(s) (3.2) (*p* < *0.05*). Moreover, the level of burnout complaints in the group with physical chronic disease(s) was significantly lower than in the group with mental chronic disease(s) (*p* < 0.05) and the group with both physical and mental condition(s) (*p* < 0.05). The mean level of work engagement was significantly higher in employees without chronic diseases (5.1), than in the group with mental chronic disease(s) (4.2) (*p* < 0.05) and the group with both physical and mental condition(s) (4.3) (*p* < 0.05), but not different from the group with physical chronic disease(s) (5.1). Furthermore, the level of work engagement in the latter group was significantly higher than in the group with mental chronic disease(s) (*p* < 0.05) and the group with both physical and mental condition(s) (*p* < 0.05).

The level of each of the four job resources (autonomy, social support by colleagues, a supportive leadership style, or an open and communicative culture) in the group with physical chronic disease(s) and the group with mental chronic disease(s) was at the same level as in the group without chronic diseases. The level of each job resource was significantly lower in the group with physical and mental chronic disease(s) than in the group without chronic diseases (*p* < 0.05) (see Table 3).

As shown in Table 4, significant correlations (*p* < 0.01) between work ability, burnout complaints, work engagement, and each of the four job resources were observed in the expected directions.

**TABLE 4:** Work ability, burnout complaints, work engagement, and job resources (autonomy, social support by colleagues, supportive leadership style, or open and communicative culture): correlations.

**Variables**	**1**	**2**	**3**	**4**	**5**	**6**	**7**
Work ability	(0.74)						
Burnout complaints	−0.579**	(0.89)					
Work engagement	0.434**	−0.781**	(0.95)				
Autonomy	0.312**	−0.362**	0.282**	(0.80)			
Social support by colleagues	0.161**	−0.322**	0.355**	0.171**	(0.74)		
Supportive leadership style	0.238**	−0.352**	0.343**	0.240**	0.351**	(0.89)	
Open and communicative culture	0.233**	−0.385**	0.380**	0.250**	0.356**	0.618**	(0.90)

Cronbach’s α’s are in parentheses. **Correlation is significant at the 0.01 level (two-tailed).

### Hypothesis Testing

The explained variances of the regression models were 22% for work ability, 32% for burnout complaints, and 27% for work engagement. Age is a predictor with a small effect size for lower work ability (β = −0.091, *p* < 0.01) and lower work engagement (β = −0.040, *p* < 0.05); however, age is no predictor for the level of burnout complaints (see Table 5).

**TABLE 5:** Summary of multiple regression analyses for variables predicting work ability, burnout complaints, and work engagement (*N* = 1951).

	**Work ability**			**Burnout complaints**			**Work engagement**		
**Variable**	***B***	***SE B***	**β**	***B***	***SE B***	**β**	***B***	***SE B***	**β**
Age	–0.004	0.001	−0.091**	0.000	0.001	–0.003	–0.004	0.002	−0.040*
Group 1—physical chronic disease(s)	–0.097	0.024	−0.085**	0.046	0.031	0.028	0.053	0.048	0.022
Group 2—mental chronic diseases(s)	–0.371	0.096	−0.092**	0.489	0.124	0.087**	–0.698	0.190	−0.084**
Group 3—physical chronic disease(s) and mental chronic diseases(s)	–0.535	0.053	−0.235**	0.671	0.069	0.212**	–0.621	0.106	−0.133**
Autonomy	0.117	0.015	0.214**	–0.157	0.020	−0.207**	0.151	0.031	0.135**
Group 1 × Autonomy	–0.011	0.025	–0.012	–0.050	0.033	–0.038	0.078	0.050	0.040
Group 2 × Autonomy	0.254	0.098	0.069*	–0.212	0.127	–0.041	0.074	0.195	0.010
Group 3 × Autonomy	0.195	0.047	0.096**	–0.150	0.060	−0.053*	0.077	0.092	0.018
Social support of colleagues	0.018	0.016	0.034	–0.133	0.021	−0.177**	0.243	0.032	0.218**
Group 1 × Social support of colleagues	–0.027	0.027	–0.027	0.064	0.035	0.047	–0.060	0.053	–0.030
Group 2 × Social support of colleagues	–0.046	0.105	–0.011	–0.128	0.135	–0.021	0.064	0.207	0.007
Group 3 × Social support of colleagues	0.048	0.047	0.026	–0.091	0.061	–0.035	0.045	0.093	0.012
Supportive leadership style	0.071	0.019	0.130**	–0.100	0.025	−0.132**	0.159	0.038	0.143**
Group 1 × Supportive leadership style	–0.023	0.032	–0.023	–0.004	0.041	–0.003	–0.062	0.063	–0.031
Group 2 × Supportive leadership style	–0.319	0.150	−0.070*	0.180	0.195	0.028	–0.220	0.298	–0.024
Group 3 × Supportive leadership style	–0.051	0.055	–0.029	0.170	0.071	0.070*	–0.312	0.108	−0.087**
Open and communicative culture	0.020	0.020	0.038	–0.115	0.025	−0.152**	0.229	0.039	0.206**
Group 1 × Open and communicative culture	0.056	0.031	0.059	–0.027	0.040	–0.020	–0.049	0.061	–0.025
Group 2 × Open and communicative culture	0.052	0.130	0.011	–0.181	0.168	–0.028	0.204	0.258	0.021
Group 3 × Open and communicative culture	–0.076	0.056	–0.041	–0.006	0.073	–0.002	–0.083	0.111	–0.022
*R*^2^	0.217			0.321			0.267		
*F*	26.159			44.535			34.292		

*Correlation is significant at the 0.05 level (two-tailed). **Correlation is significant at the 0.01 level (two-tailed).

The first set of hypotheses was partly confirmed. All three chronic disease groups were associated with lower work ability (β’s −0.085, −0.092, and −0.235, respectively; *p* < 0.01). However, groups 2 and 3 (both groups with mental conditions, with and without physical chronic diseases) were related to higher burnout complaints (β’s 0.087 and 0.212, respectively; *p* < 0.01) and to lower work engagement (β’s −0.084 and −0.133, respectively; *p* < 0.01), but this was not the case for the group with exclusively physical chronic diseases (see Table 5). In other words, H1a is completely supported and H1b and H1c are partly supported.

The analyses of the second set of hypotheses regarding the four job resources demonstrates that only autonomy and a supportive leadership style were associated in the expected directions with work ability, burnout complaints, and work engagement. Social support by colleagues and an open and communicative organizational culture were only associated with burnout complaints and work engagement as expected, but not associated with work ability (see Table 5). In other words, H2a and H2c are completely supported and H2b and H2d are partially supported.

The analyses of the third set of hypotheses regarding the possible moderation by the four job resources of the association between the chronic disease groups with the three indicators of occupational well-being resulted in some significant results for autonomy and for a supportive leadership style. However, no moderation was found for social support of colleagues, nor for an open and communicative organizational culture.

**FIGURE 1:**
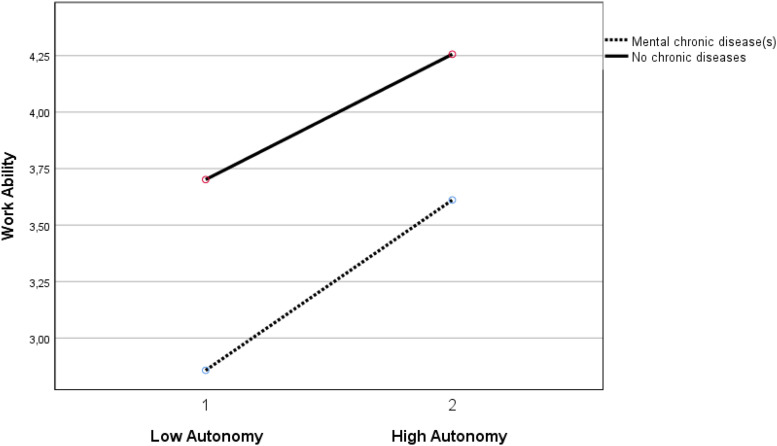
Moderation effect between autonomy and the group with mental chronic diseases versus the group without chronic diseases on work ability. Autonomy 1 is ≤1 standard deviation below mean. Autonomy 2 is ≥1 standard deviation above mean.

**FIGURE 2:**
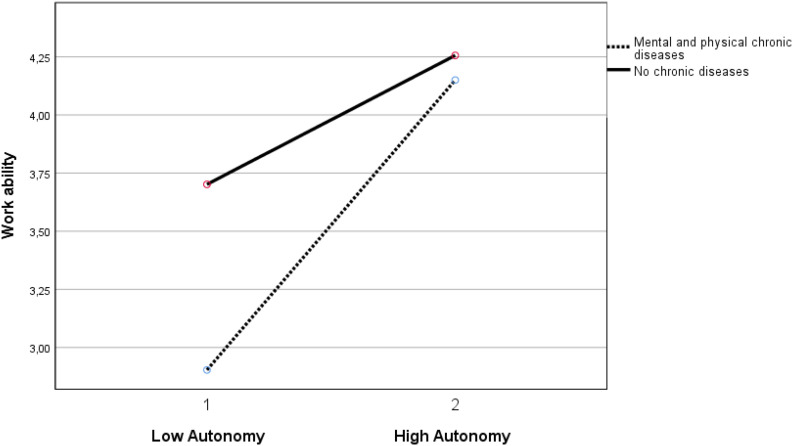
Moderation effect between autonomy and the group with mental and physical chronic diseases versus the group without chronic diseases on work ability. Autonomy 1 is ≤1 standard deviation below mean. Autonomy 2 is ≥1 standard deviation above mean.

**FIGURE 3:**
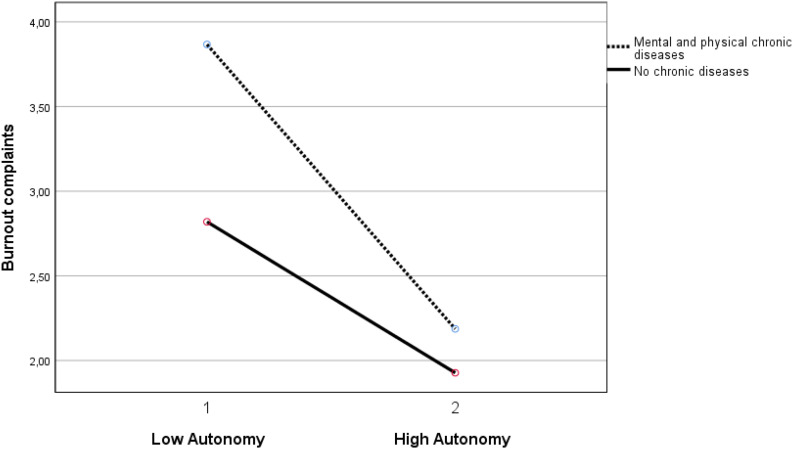
Moderation effect between autonomy and the group with mental and physical chronic diseases versus the group without chronic diseases on burnout complaints. Note: Autonomy 1 is ≤1 standard deviation below mean. Autonomy 2 is ≥1 standard deviation above mean.

Autonomy buffered the negative relationship of both the group with mental chronic disease(s) (β = 0.069, *p* < 0.05) and the group with both mental and physical chronic diseases (β = 0.096, *p* < 0.01) with work ability (see Figures 1, 2). Autonomy also buffered the positive relationship of the group with physical and mental chronic disease(s) (β = −0.053, *p* < 0.05) with burnout complaints (see Figure 3).

**FIGURE 4:**
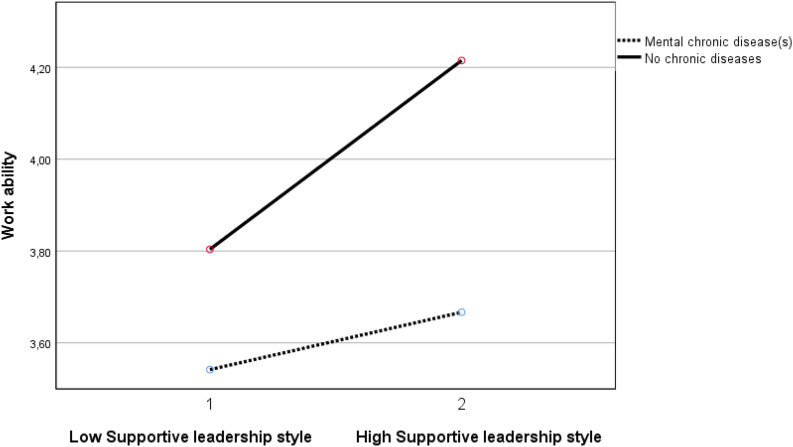
Moderation effect between supportive leadership style and the group with mental chronic diseases versus the group without chronic diseases on work ability. Supportive leadership style 1 is ≤1 standard deviation below mean. Supportive leadership style 2 is ≥1 standard deviation above mean.

**FIGURE 5:**
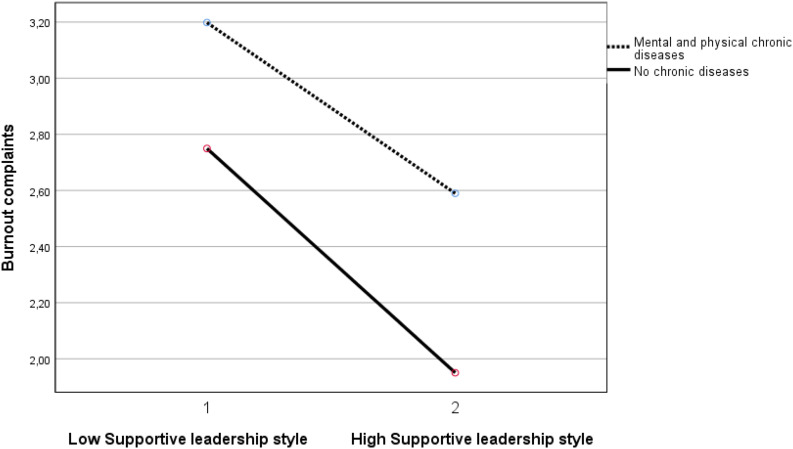
Moderation effect between supportive leadership style and the group with mental and physical chronic diseases versus the group without chronic diseases on burnout complaints. Supportive leadership style 1 is ≤1 standard deviation below mean. Supportive leadership style 2 is ≥1 standard deviation above mean.

**FIGURE 6:**
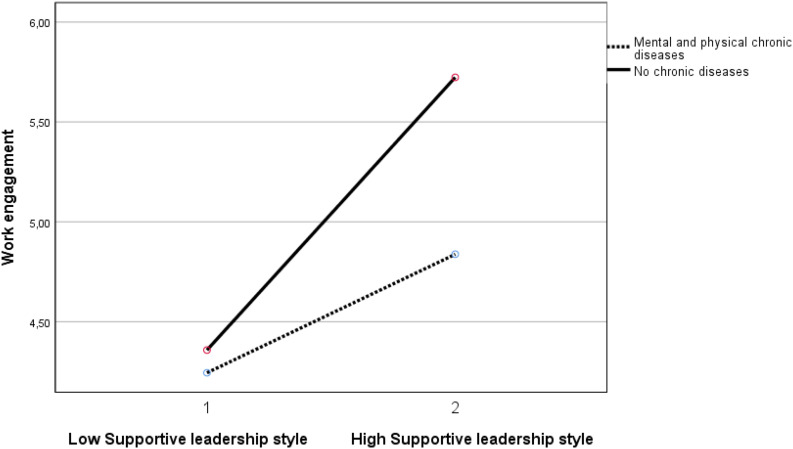
Moderation effect between supportive leadership style and the group with mental and physical chronic diseases versus the group without chronic diseases on work engagement. Supportive leadership style 1 is ≤1 standard deviation below mean. Supportive leadership style 2 is ≥1 standard deviation above mean.

A supportive leadership style also demonstrates three significant moderation effects, namely in (1) the negative relationship between the group with mental chronic disease(s) with work ability (β = −0.070, *p* < 0.05), (2) the positive relationship between the group with physical and mental chronic disease(s) with burnout complaints (β = 0.070, <0.05), and (3) the negative relationship between the group with physical and mental chronic disease(s) with work engagement (β = −0.087, < 0.01). The results indicate that a supportive leadership style is less beneficial for the employees with mental chronic diseases than for the employees without chronic diseases. In other words, the group without chronic diseases demonstrates an interaction effect by a supportive leadership style resulting in a larger increase in work ability and in work engagement and in a larger decrease of burnout complaints, than the abovementioned groups with chronic diseases (see Figures 4–6). See Table 5 for all results. In short, the results supported H3a and H3c partially, and H3b and H3d were not supported.

## Discussion and Conclusion

The expectation that the presence of one or more chronic diseases was associated with lower work ability is met. So, employees with a physical or a mental chronic disease, or both, are at risk of experiencing a lower work ability. This is in line with other studies (Koolhaas et al., 2013; Leijten et al., 2014; Kadijk et al., 2018). The group with mental and physical chronic diseases is the most vulnerable as the effect size for this group is at the highest level (between small and medium). Moreover, age is also negatively associated with work ability as demonstrated in other studies (van den Berg et al., 2008); however, this association is small. The expectation that the presence of one or more chronic diseases was associated with higher burnout complaints and with a lower work engagement was partly met, as this was not the case when only physical chronic disease(s) were involved. In the case that mental chronic diseases are involved, higher burnout complaints and lower work engagement were present, as are also reported in other studies. Concerning burnout complaints, any possible relations with depression, a highly prevalent mental condition, sharing its etiology with burnout (Shirom et al., 2006), might play a role. This also might clarify that the group with exclusively physical chronic diseases demonstrates no association with higher burnout complaints. However, the latter is not in line with other studies, like in the Finnish nationwide population studies. Higher burnout was reported among women with coronary heart disease (Hallman et al., 2003), and among women with musculoskeletal diseases and men with cardiovascular diseases (Honkonen et al., 2006). Possibly, the comparability between studies is affected because the study samples have different profiles with regard to the combination of various types of physical chronic diseases and also because the present study included a specific segment of the labor market. With regard to the absence of an association of physical chronic diseases with lower work engagement in the present study, there are very few studies to make comparisons with. Further, in the present study, the level of work engagement is a little lower with higher age, which is in contrast to several studies indicating workers are more engaged as they age (Kim and Kang, 2017). In short, the results demonstrate that the occupational well-being of the workers with mental chronic diseases is vulnerable, as their chronic disease is associated with a higher level of burnout complaints and a lower level of work engagement.

Not all associations of each of the four job resources with each of the three measures of occupational well-being are as expected. The associations of autonomy and a supportive leadership style with each of the three measures of occupational well-being are as expected, with the highest effect size for autonomy predicting higher work ability and lower burnout complaints. The associations of social support of colleagues and an open and communicative organizational culture with each of the three measures of occupational well-being are not as expected, as these two job resources are not associated with work ability. This is an important finding as studies on the association between these two job resources and work ability are scarce.

Regarding any possible moderation effects, only autonomy and a supportive leadership style demonstrate significant results in the associations between the groups that include participants with mental chronic diseases. However, the results for these two job resources point in different directions. The buffering effect of autonomy is in line with several previous studies and as we expected. So, autonomy is an important job resource to alleviate the associations of chronic diseases with less favorable occupational well-being. However, the moderation effects of a supportive leadership style are surprising, as these indicate a supportive leadership style to be of less benefit for occupational well-being among employees with mental chronic diseases (with or without physical chronic diseases) than among employees without chronic diseases. A supportive leadership style does only slightly buffer the negative relationship of a mental chronic disease with lower work ability, higher burnout complaints, or lower work engagement. Moreover, the total level of the experienced supportive leadership style in the group with physical and mental comorbidities (see Table 3) is significantly lower than in all the other groups. Because of the cross-sectional design, we can only guess about the causes, but an explanation might be that because of their poorer well-being, employees with more severe mental chronic diseases receive more support from their supervisor or manager than the employees with less severe mental chronic diseases. Employees with a mental disorder especially are faced with stigma (van Vuuren et al., 2017; Brouwers et al., 2019; Brouwers, 2020), and perhaps more reluctant to share their chronic disease with their supervisor or manager. As not all employees tell their supervisor or manager about their mental chronic disease, it might as well be possible that for many employees their mental chronic disease is not known by the manager or supervisor as long it is not severe enough to interfere noticeably with work functioning. It is also possible that in the case that a supervisor or manager does know about the mental chronic disease, he or she keeps more emotional distance than with the group without mental chronic diseases. Furthermore, employees with mental chronic diseases might experience less support from their supervisor or manager as long as their functioning is acceptable, up to the situation that problems with occupational well-being are shared with their supervisor or manager and become visible in the workplace, generating more support from their supervisor or manager.

The two other job resources, social support of colleagues and an open and communicative organizational culture, demonstrate no moderation at all. Unfortunately, studies into this subject are scarce. Studies among workers past cancer diagnosis reported the buffering effects of social support of colleagues, as well as a better social climate at work, with regard to work ability in the population of workers past cancer diagnosis (Taskila and Lindbohm, 2007; Taskila et al., 2007). However, this is a specific group and only concerns work ability.

Although the group with exclusively mental chronic diseases is relatively small (*N* = 36, 1.8%), their mean age (42.1 years) is significantly lower as the group with exclusively physical chronic diseases (47.9 years). The mean age of the group with comorbidity of physical and mental chronic diseases is 47.5 years. This implies that many employees with chronic diseases will have around two decades of employment ahead. As the level of all four job resources among this comorbidity group is experienced significantly lower than among the group without chronic diseases, the group with physical and mental chronic disease(s) needs particular attention with respect to the experienced level of the job resources.

In general, the research field concerning chronic diseases, the experience of job resources, and the association with work ability, burnout complaints, and work engagement still seems to be a niche. Nevertheless, the present results raise concerns with regard to the occupational well-being of the employees with mental chronic diseases, with or without physical chronic diseases. In addition, there might be a potential to increase their occupational well-being by offering job resources, especially more autonomy.

### Limitations

It is important to notice that the population of the present study, consisted of a specific sub-group of Dutch employees, namely, employees in educational and (semi) governmental organizations. These employees might have a specific profile compared to the Dutch nationwide employed population. However, a comparison with the general employed population was not the aim, and the focus was on the associations within the group of participants. Furthermore, self-reported measures might be biased, and hence offer an inadequate indication of the level of the job resources offered in the workplace. However, one’s own interpretation of the job resources causes the action, as formulated in the Thomas theorem (Thomas and Thomas, 1928, p. 572): “If men define situations as real, they are real in their consequences.” In other words, self-reported measures are necessary to find out how employees experience their own situation. Additionally, no causal inferences can be made because of the cross-sectional nature of the study.

### Practical Implications

This study demonstrated that autonomy is an important target for interventions to enhance work ability and work engagement and to reduce burnout complaints among employees with mental chronic diseases. As autonomy covers many possibilities in the context of work and can range from making decisions about one’s own work breaks, to making decisions on work procedures, this job resource offers many opportunities. These possibilities should be elaborated between an employer and the employee as much as possible. However, this probably also requires a supportive leadership style, and the results in the present study regarding this job resource are unexpectedly less favorable. So, to imbed more autonomy, the experience of a supportive leadership style also needs attention.

Furthermore, the choice of workers not to disclose chronic diseases can be understandable (Brouwers et al., 2019; Brouwers, 2020); however, as a consequence, this prevents extra attention and effort by the supervisor or manager in managing more autonomy for these employees. Nevertheless, in work situations where an employee experiences a low level of autonomy, especially in the case that chronic diseases are disclosed, a very important question is what possibilities might be present to enhance the level of autonomy.

Also, supervisors, line managers, and human resource management should work together in this process, as the perspectives on the role of specific job resources have demonstrated to be different between certain positions (Haafkens et al., 2011) and a collaboration will present a broader perspective on options in the context of the work situation.

Furthermore, involving the employees in exploring possibilities can also generate important workable solutions and will be an opportunity for more autonomy and experiencing more support in the context of work. It is important to make use of experiential experts of all the parties involved.

To conclude, interventions focusing on autonomy offer opportunities to reinforce work ability and work engagement and to reduce burnout complaints among employees with mental chronic diseases, with or without physical chronic diseases.

## Data Availability Statement

The datasets generated for this study will not be made publicly available. The data that support the findings are available from Loyalis Knowledge & Consult but restrictions apply to the availability of these data, which were used under license for the current study, and so are not publicly available. The data are however available from the authors upon reasonable request and with permission of Loyalis Knowledge & Consult.

## Ethics Statement

Ethical review and approval was not required for the study on human participants in accordance with the local legislation and institutional requirements. The patients/participants provided their written informed consent to participate in this study.

## Author Contributions

IB, WV, and TV developed the study design. TV was responsible for the data collection. The data analysis was prepared by IB with support from TV and WV. IB wrote the first draft of the manuscript, and the later drafts of the manuscript were adjusted by all three authors in collaboration. All authors read and approved the submitted version.

## Conflict of Interest

The authors declare that the research was conducted in the absence of any commercial or financial relationships that could be construed as a potential conflict of interest.
